# Nonstoichiometry, Defect Chemistry and Oxygen Transport in Fe-Doped Layered Double Perovskite Cobaltite PrBaCo_2−*x*_Fe*_x_*O_6−δ_ (*x* = 0–0.6) Membrane Materials

**DOI:** 10.3390/membranes12121200

**Published:** 2022-11-28

**Authors:** Ivan L. Ivanov, Petr O. Zakiryanov, Vladimir V. Sereda, Maxim O. Mazurin, Dmitry A. Malyshkin, Andrey Yu. Zuev, Dmitry S. Tsvetkov

**Affiliations:** Institute of Natural Sciences and Mathematics, Ural Federal University, 19 Mira St., Ekaterinburg 620002, Russia

**Keywords:** point defects, chemical diffusion, self-diffusion, surface exchange, activation energy, defect chemistry, mixed conductor

## Abstract

Mixed conducting cobaltites PrBaCo_2−*x*_Fe*_x_*O_6−δ_ (*x* = 0–0.6) with a double perovskite structure are promising materials for ceramic semi-permeable membranes for oxygen separation and purification due to their fast oxygen exchange and diffusion capability. Here, we report the results of the detailed study of an interplay between the defect chemistry, oxygen nonstoichiometry and oxygen transport in these materials as a function of iron doping. We show that doping leads to a systematic variation of both the thermodynamics of defect formation reactions and oxygen transport properties. Thus, iron doping can be used to optimize the performance of mixed conducting oxygen-permeable double perovskite membrane materials.

## 1. Introduction

Since the discovery of fast oxide ion transport in the A-site-ordered layered double perovskite cobaltites *RE*BaCo_2_O_6−δ_ (*RE* = rare-earth element) [1], they have received a lot of attention as suitable materials for various electrochemical devices, including oxygen-semi-permeable membranes [2,3,4]. In addition, high mixed oxide-ion and electronic conductivity coupled with the catalytic activity towards the oxygen reduction reaction suggested *RE*BaCo_2_O_6−δ_ cobaltites as promising cathode materials for both oxide-ion and proton-conducting solid oxide fuel cells [2,3,4,5]. As a result, a number of studies describing various aspects of practical applications of double perovskites have been produced, but the understanding of the oxygen transport in these oxides, despite being directly responsible for their performance, remains underdeveloped. There is some scatter in the reported activation energies, values of the oxygen self-diffusion coefficient and surface exchange coefficient even for the undoped cobaltites [5,6,7,8,9,10,11,12,13], whereas for the doped ones, such measurements are exceptionally rare [7]. Furthermore, although the mechanism of oxygen diffusion in the A-site ordered layered double perovskites is known [6,14,15,16,17,18,19,20,21,22] and may be described as the oxygen diffusion within the CoO_2−_layers through oxygen vacancies coming from the rare-earth layers, the detailed understanding of the dopant-induced changes is also lacking. The same scarcity of systematic reports can be noted about the defect chemistry which, with the rare exceptions [23,24,25,26,27,28], has not been studied yet for doped double perovskites.

Substituting iron for cobalt in various perovskite-type cobaltites is a widely used strategy for lowering their thermal expansion coefficient (TEC) [29,30] and increasing their thermodynamic stability [31,32,33]. While there are data indicating that moderate iron content in *RE*BaCo_2−*x*_Fe*_x_*O_6−δ_ does not lower their TEC significantly [30,34], their stability seems to increase [31,32,33]. In addition, some studies report that iron doping enhances the electrochemical performance of double perovskite cobaltites as cathodes in solid oxide fuel cells [30] and oxygen evolution reaction catalysts [33].

Therefore, in this work we report the results of the detailed study of an interplay between the defect chemistry, oxygen nonstoichiometry and oxygen transport in the double perovskites PrBaCo_2−*x*_Fe*_x_*O_6−δ_ (PBCF) with *x* = 0–0.6 as a function of the iron doping. We show that doping leads to a systematic variation of both the thermodynamics of defect formation reactions and oxygen transport properties, and that it can be used to optimize the performance of these mixed conducting oxygen-permeable membrane materials.

## 2. Experimental

The powder samples of PrBaCo_2_O_6−δ_ (PBC), PrBaCo_1.8_Fe_0.2_O_6−δ_ (PBCF2), PrBaCo_1.6_Fe_0.4_O_6−δ_ (PBCF4) and PrBaCo_1.4_Fe_0.6_O_6−δ_ (PCBF6) were synthesized via the glycerol-nitrate route using Pr_6_O_11_ (purity 99.99 wt.%, Lanhit, Moscow, Russia), BaCO_3_ (purity 99.99 wt.%, Lanhit, Russia), FeC_2_O_4_·2H_2_O (purity 99.2 wt.%, Vekton, Moscow, Russia) and Co as the precursors. Metallic cobalt was obtained reducing Co_3_O_4_ (purity 99.97 wt.%, MCP HEK GmbH, Lübeck, Germany) in a hydrogen flow at 600 °C. Pr_6_O_11_ and BaCO_3_ were pre-annealed at 450 °C in air to remove absorbed H_2_O and CO_2_. Stoichiometric quantities of the precursors were dissolved in concentrated HNO_3_ (purity 99.99 wt.%, Vekton, Russia), mixed with glycerol (purity 99.5 wt.%, Vekton, Russia), whose amount was slightly greater than needed to reduce all the nitrates in the solution to the corresponding oxides and N_2_, and then heated on a hot plate until complete evaporation and pyrolysis had occurred. The as-obtained powder samples were calcined at 900 °C, 1000 °C and 1100 °C in air with intermediate regrindings; the dwell time at each temperature was 10 h.

The phase purity of the samples prepared accordingly was confirmed by powder X-ray diffraction method (XRD). The XRD patterns were obtained with a 7000S (Shimadzu, Kyoto, Japan) X-ray diffractometer using Cu Kα radiation.

In situ high-temperature XRD measurements (HT XRD) were carried out using the 7000S (Shimadzu, Japan) diffractometer equipped with a high temperature chamber HTK 1200N (Anton Paar, Graz, Austria). Oxygen partial pressure (*p*O_2_) around the sample was controlled by a potentiometric sensor made of yttria stabilized zirconia (YSZ) and maintained by mixing gaseous N_2_ and O_2_ in appropriate ratios using mass flow controllers RRG12 (Eltochpribor, Moscow, Russia). Before the measurements, the samples were equilibrated at each selected *p*O_2_ and *T* for, at least, 6 h.

The ceramic samples for conductivity measurements were prepared by uniaxially pressing the single-phase powders into rectangular bars of 30 × 4 × 4 mm^3^ at 20 MPa and sintering the bars at 1250 °C for 24 h in air. The relative density of the samples prepared accordingly was found to be 95%. The chemical composition of the as-prepared ceramic bars was examined using a VEGA 3 scanning electron microscope (Tescan, Brno, Czech Republic) equipped with an Ultim Max 40 detector (Oxford Instruments, Abingdon, UK). The results are summarized in Table S1 and Figures S1 and S2. (see Supplementary Materials); the measured and nominal compositions of the samples were found to be in good agreement.

**Figure 1:**
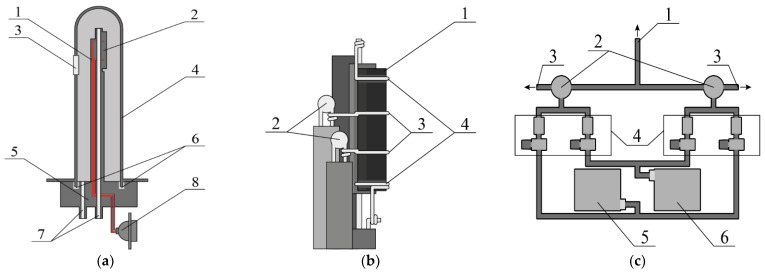
Schematic drawings of the functional parts of the conductivity relaxation setup: (**a**) The measurement cell in the YSZ tube: 1—alumina sample holder, 2—sample, 3—Pt electrodes, 4—YSZ tube, 5—aluminum flange, 6—silicon rubber O-ring, 7—gas inlet and outlet, 8—electrical connector. (**b**) Enlarged view of the upper part of the sample holder: 1—sample, 2—type S thermocouples, 3, 4—Pt wires for conductivity measurements. (**c**) Gas mixing unit: 1—gas tube (304 stainless steel) leading to the measurement cell, 2—3-way electrical valves, 3—gas bypass outlets, 4—mass flow controllers, 5—dry air compressor, 6—nitrogen generator.

The electrical conductivity relaxation (ECR) measurements were carried out by means of a homemade apparatus. Its schematic representation is shown in Figure 1. The ECR apparatus consists of a custom-built high temperature furnace with a sample holder (see Figure 1a,b) placed inside. The sample chamber, supplied with gas inlet and outlet, is isolated from the ambient atmosphere by a gas-tight YSZ tube. The porous platinum electrodes, applied to the YSZ electrolyte, form a potentiometric *p*O_2_ sensor for monitoring the oxygen partial pressure around the sample. Simultaneous temperature measurement and regulation, *p*O_2_ measurements, and 4-probe DC conductivity measurements are performed with a Zirconia 318 (UrFU, Ekaterinburg, Russia) universal controller. A homemade computer-controlled 4-channel gas mixing unit (see Figure 1c) employing the RRG12 (Eltochpribor, Russia) mass flow controllers and 3-way-electrical valves (SMC, Tokyo, Japan) allows maintaining and rapidly switching the *p*O_2_ around the sample to induce the conductivity relaxation. The following stepwise changes of log(*p*O_2_/atm) were implemented: −0.678 → −0.88 → −1.14 → −1.37 → −1.55 → −1.87 → −2.20 → −2.84 → −3.25 (log is the logarithm with base 10). The 3-way valves in the mixer effectively switch the fixed-rate gas flows, directing them either to the bypass or to the measurement cell. This eliminates the transient effects occurring upon changing the setpoints in the mass-flow controllers, which would not be possible in a simpler gas mixer setup. The volume of the sample chamber is about 20 cm^3^, whereas the gas flow rate used in this work was 150 cm^3^·min^−1^, which means the *p*O_2_ around the sample can be changed during the experiment in a matter of a few seconds. The dry air and nitrogen supplied to the gas mixing unit were produced on site using specialized dry-air compressor (KSV-6/500, NPP Himelektronika, Moscow, Russia) and nitrogen generator (GChA-21D, NPP Himelektronika, Russia), respectively.

The sample’s electrical conductivity measured after each *p*O_2_ change was normalized according to Equation (1):

(1) $$\sigma_{\mathrm{norm}}=\frac{\sigma_{t}-\sigma_{0}}{\sigma_{\infty }-\sigma_{0}}$$

where *σ_t_*, *σ*_0_ and *σ*_∞_ are the conductivity at time *t*, the equilibrium conductivity before the *p*O_2_ change and the equilibrium conductivity after the *p*O_2_ change, respectively. The examples of the time-dependent normalized conductivity calculated with Equation (1) from the relaxation measurement results are shown in the Supplementary, Figures S3 and S4.

The surface exchange coefficient, *k*_chem_, and the bulk oxygen chemical diffusion coefficient, *D*_chem_, were estimated by fitting the solution of Fick’s second law [35,36,37], Equation (2), to the resulting relaxation curves *σ*_norm_ vs. time.

(2) $$\sigma_{\mathrm{norm}}=1-\sum_{m}^{\infty }\sum_{n}^{\infty }\sum_{p}^{\infty }\frac{2L_{\beta }^{2}\exp\left(\frac{-\beta_{m}^{2}D_{\mathrm{chem}}t}{x^{2}}\right)}{\beta_{m}^{2}\left(\beta_{m}^{2}+L_{\beta }^{2}+L_{\beta }\right)}\frac{2L_{\gamma }^{2}\exp\left(\frac{-\gamma_{n}^{2}D_{\mathrm{chem}}t}{y^{2}}\right)}{\gamma_{n}^{2}\left(\gamma_{n}^{2}+L_{\gamma }^{2}+L_{\gamma }\right)}\frac{2L_{\beta }^{2}\exp\left(\frac{-\theta_{p}^{2}D_{\mathrm{chem}}t}{z^{2}}\right)}{\theta_{p}^{2}\left(\theta_{p}^{2}+L_{\theta }^{2}+L_{\theta }\right)}$$

(3) $$L_{\beta }=x\frac{k_{\mathrm{chem}}}{D_{\mathrm{chem}}};\;L_{\gamma }=y\frac{k_{\mathrm{chem}}}{D_{\mathrm{chem}}};\;L_{\theta }=z\frac{k_{\mathrm{chem}}}{D_{\mathrm{chem}}}$$

where *t* is time; 2*x*, 2*y*, 2*z* are the dimensions of the sample, and *β_m_*, *γ_n_*, *ϴ_p_* are the positive, non-zero roots of the following equations:

(4) $$\beta_{m}\tan\left(\beta_{m}\right)=L_{\beta };\;\gamma_{n}\tan\left(\gamma_{n}\right)=L_{\gamma };\;\theta_{p}\tan\left(\theta_{p}\right)=L_{\theta }$$

The values of *β_m_*, *γ_n_*, and *ϴ_p_* are computed iteratively as described in [37] during the fitting after each optimization step. The oxygen self-diffusion coefficient, *D*_O_, and the surface exchange coefficient, *k*, were calculated from the as-determined chemical ones, *D*_chem_ and *k*_chem_, according to Equations (5) and (6):

(5) $$D_{O}=\frac{D_{\mathrm{chem}}}{\gamma }$$

and

(6) $$k=\frac{k_{\mathrm{chem}}}{\gamma }$$

where $\gamma =\frac{1}{2}{\left(\frac{\partial \ln p_{O_{2}}}{\partial \ln c_{O}}\right)}_{T}$ is the thermodynamic enhancement factor and *c*_O_ is the concentration of oxygen in the corresponding oxide. The values of *γ* were found from the isothermal *p*O_2_ dependencies of the oxygen content measured in this work for all the studied samples of double perovskites.

The ECR measurements were carried out both in the direction of decrease and increase in *p*O_2_. The resulting parameters *D*_chem_ and *k*_chem_ estimated as mentioned above did not show any dependence on the direction of *p*O_2_ change and were averaged.

In order to obtain the *p*O_2_–*T*–δ data necessary for estimation of the thermodynamic enhancement factor, γ, and analysis of the defect structure of the double perovskites PrBaCo_2−*x*_Fe_*x*_O_6−δ_, the relative change in their oxygen nonstoichiometry (Δδ) was measured in the wide ranges of *T* and *p*O_2_ by two independent techniques: coulometric titration (CT) and thermogravimetry (TG), described in detail elsewhere [38]. The CT measurements were carried out in a home-made setup [38]. The TG measurements were performed using a RuboTHERM Dyntherm LP-ST thermobalance (TA Instruments, New Castle, DE, USA). Powder samples weighing ~0.5 g were used for both TG and CT measurements.

As the instantaneous equilibration criteria during CT procedure the slope of the $\log\left(pO_{2},\;\mathrm{atm}\right)=f\left(time,s\right)$ curve was used. It was monitored during each titration step. The state of the titration cell was regarded as “stationary” if this slope did not exceed 10^−6^ for a few hours, which gives negligible *p*O_2_ change speed.

In the TG experiment the equilibrium state of the sample was assumed when its weight ceased to change. In addition, all the TG and CT measurements were performed so as to ensure that the results are reproducible and correspond to the true equilibrium state, the main sign of which is the reversibility, i.e., the independence of the results on the direction of the *p*O_2_ change. To check the reproducibility, the measurements were carried out in the directions of both the increase and the decrease in the *p*O_2_. The relative nonstoichiometry obtained did not depend on the mode of the experiment and, hence, was assumed to correspond to the true equilibrium state of the sample.

The relative change in the oxygen nonstoichiometry obtained by TG or CT was then recalculated to the absolute scale (δ) using the absolute oxygen content, determined by means of direct sample reduction in the flow of pure hydrogen at 1200 °C in the STA 409 PC (Netzsch, Selb, Germany) thermobalance [38].

## 3. Results and Discussion

### 3.1. Phase Composition and Crystal Structure of the Double Perovskites PBCF

**Figure 2:**
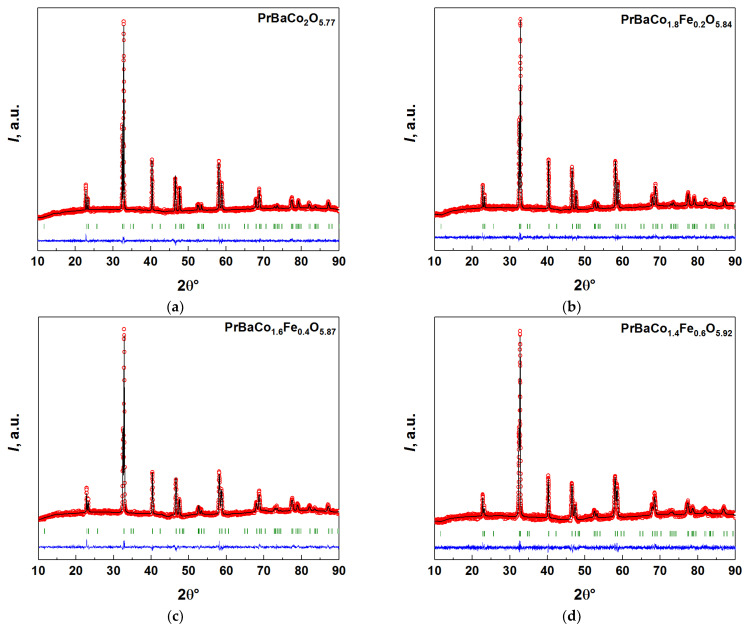
The XRD patterns of the as-prepared, slowly (2 °C·min^−1^) cooled in air, samples of PBCFs: (**a**) PBC, (**b**) PBCF2, (**c**) PBCF4, (**d**) PBCF6: red circles—experimental data, black line—calculated pattern, green vertical lines—positions of allowed Bragg reflections, blue line at the bottom—difference between the observed and calculated patterns.

The XRD patterns of the as-prepared, slowly (2 °C·min^−1^) cooled in air, samples of PBCF are shown in Figure 2. All the samples were confirmed to be single-phase in agreement with the previous studies [4,7,39,40]. Their XRD patterns were indexed using the *P*4*/mmm* space group. The lattice parameters refined by the Rietveld method are given in Table S1. As seen, they are in good agreement with those reported earlier [4,7,39,40]. At the same time, in contrast to [40] where PBCF samples with *x* = 0.4 and 0.6 were found to possess cubic ‘simple’ perovskite crystal structure, in the present study all the PBCF samples in the range of 0 ≤ *x* ≤ 0.6 were obtained as double perovskites with tetragonal crystal structure. The main reason behind this discrepancy seems to be the thermal treatment of the samples during their preparation. Indeed, the highest calcination temperature used for the synthesis in [40] was 950 °C, as compared with 1100 °C employed by us. Grimaud et al. [7] also came to the same conclusions.

In situ HT XRD was used to study the phase composition and crystal structure of the PBCF samples in the wide range of *T* and *p*O_2_ employed for ECR measurements. As a result (see Supplementary, Figures S5–S8) tetragonal double perovskite structure was confirmed for all the studied samples in the ranges 550 ≤ *T*/°C ≤ 1000 and 10^−3^ ≤ *p*O_2_/atm ≤ 0.21, in full agreement with the previous reports [15,22,40]. However, at temperatures lower than 550 °C and *p*O_2_ ≤ 10^−3^ atm the formation of orthorhombic crystal structure with the *Pmmm* space group was observed. Nevertheless, the range of *T* and *p*O_2_ corresponding to the tetragonal crystal structure is typical of the operation of oxygen separating semi-permeable membranes [2]. For this reason, all the ECR and nonstoichiometry measurements in this work were carried out under these conditions on the PBCF double perovskite samples belonging to the *P*4*/mmm* space group.

### 3.2. Oxygen Content in PBCF and Their Defect Chemistry

**Figure 3:**
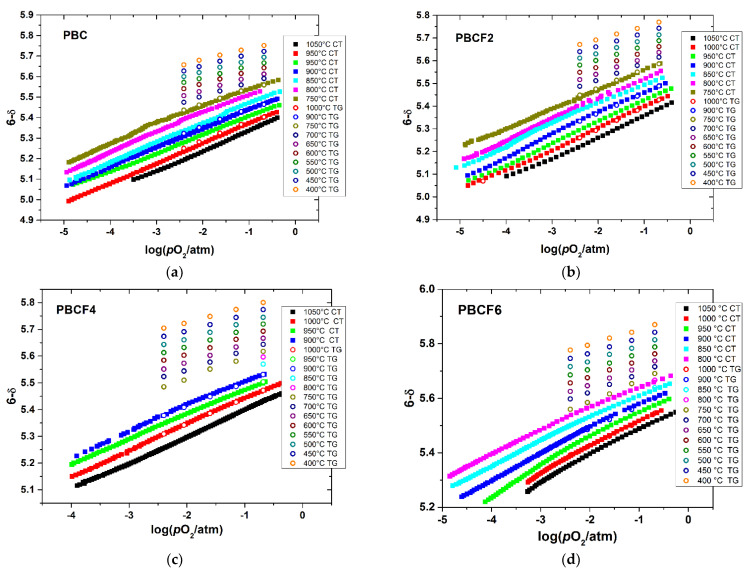
The oxygen content in PBCF as a function of *T* and *p*O_2_: (**a**) PBC, (**b**) PBCF2, (**c**) PBCF4, (**d**) PBCF6: TG—results of thermogravimetry, CT—results of coulometric titration.

The measured oxygen content, 6−δ, in the double perovskites PBCFs is shown as a function of *p*O_2_ and temperature in Figure 3. As seen, the oxygen content varies in the wide range from ~5 to ~5.8. It changes smoothly as a function of both *p*O_2_ and *T*, indicating the absence of phase transitions in the studied double perovskites, in accordance with the results of in situ HT XRD discussed above. The oxygen nonstoichiometry grows with increasing temperature and decreasing *p*O_2_. It also follows from Figure 3 that oxygen content values determined by coulometric titration and thermogravimetry coincide with each other quite well. Such coincidence indicates in favor of the reliability and reversibility of the obtained oxygen nonstoichiometry data.

The defect structure of PBCFs was analyzed, first, using the quasichemical model successfully employed previously for a number of undoped double perovskites *RE*BaCo_2_O_6−δ_ (*RE* = La, Pr, Nd, Sm, Eu, Gd, Y) [32,41]. It is based on the three quasichemical reactions, which are presented in Table 1 (reactions (1)–(3)), describing the oxygen exchange with surrounding atmosphere, the preferential localization of oxygen vacancies in the rare-earth-element layers and the cobalt disproportionation. However, as the cobalt sublattice of PBCF contains potentially redox-active iron cations, the model should be modified. Two additional processes may be considered, namely, the iron disproportionation:

(7) $$2{\mathrm{Fe}}_{\mathrm{Co}}^{\times }={\mathrm{Fe}}_{\mathrm{Co}}^{\prime }+{\mathrm{Fe}}_{\mathrm{Co}}^{•}$$

and the electron exchange between the iron and cobalt ions:

(8) $${\mathrm{Fe}}_{\mathrm{Co}}^{\times }+{\mathrm{Co}}_{\mathrm{Co}}^{\prime }={\mathrm{Fe}}_{\mathrm{Co}}^{\prime }+{\mathrm{Co}}_{\mathrm{Co}}^{\times }$$

where ${\mathrm{Co}}_{\mathrm{Co}}^{\times }$, ${\mathrm{Co}}_{\mathrm{Co}}^{\prime }$, ${\mathrm{Fe}}_{\mathrm{Co}}^{\times }$, ${\mathrm{Fe}}_{\mathrm{Co}}^{•}$ and ${\mathrm{Fe}}_{\mathrm{Co}}^{\prime }$ are Co^3+^, Co^2+^, Fe^3+^, Fe^4+^ and Fe^2+^-cations in the Co-sublattice, respectively (please note that hereinafter Kröger-Vink notation is employed for point defects designation and, as in our previous works [32,41], *RE*CoO_3_ was used as a reference crystal).

Taking into account that Fe^3+^ disproportionation is characterized by a much larger positive enthalpy value (>100 kJ·mol^−1^ [42,43]) as compared to the disproportionation of Co^3+^ (enthalpy~30–40 kJ·mol^−1^ [41]), it would be safe to neglect the contribution of Equation (7) to the equilibrium of point defects in the PBCF studied in this work. As for the reaction Equation (8), its equilibrium—due to the higher electronegativity of Co as compared to Fe—is expected to be shifted to ${\mathrm{Fe}}_{\mathrm{Co}}^{\times }$ and ${\mathrm{Co}}_{\mathrm{Co}}^{\prime }$. However, since the thermodynamics of the reaction Equation (8) is unknown a priori, it is difficult to estimate the extent of this shift. Therefore, the quasichemical reaction described by Equation (8) was included in the defect structure model. The equilibrium constants of these reactions together with the charge balance and mass balance conditions form the set of equations (see, for example, [41]) which was solved with respect to the concentrations of the defect species involved. The model function *p*O_2_ = *f*(*T*, δ, $\Delta H_{i}^{○}$, $\Delta S_{i}^{○}$) was obtained as a result, where $\Delta H_{i}^{○}$ and $\Delta S_{i}^{○}$ are the standard enthalpies and entropies of the quasichemical reactions. Fitting the model function to the *p*O_2_–*T*–δ-datasets measured for all the PBCFs yields large (~100 kJ·mol^−1^) enthalpy and small entropy around 0 J·mol^−1^·K^−1^ for the reaction Equation (8). This results in the very low value of the equilibrium constant *K*_8_ << 1 and, hence, the equilibrium of Equation (8) is almost entirely shifted to ${\mathrm{Fe}}_{\mathrm{Co}}^{\times }$ and ${\mathrm{Co}}_{\mathrm{Co}}^{\prime }$. Therefore, similarly to the iron disproportionation, Equation (7), the electron exchange between iron and cobalt ions, Equation (8), almost does not influence the defect chemistry of PBCFs and may be neglected.

It directly follows from the above considerations that iron in the cobalt sublattice of PBCF samples studied in this work is predominantly in the oxidation state +3. This conclusion is quite consistent with the results of Mössbauer spectroscopy [7,44,45] and in situ high-resolution electron energy loss spectroscopy [46]. Therefore, for further modelling, the oxidation state of iron was fixed as +3. This assumption simplified the model, since only three quasichemical reactions were retained for further analysis, and, quite predictably, did not worsen the quality of the fit. In fact, the as-obtained model 1 is almost the same as that used previously for the undoped double perovskites *RE*BaCo_2_O_6−δ_ [32,41]. The only difference consists in the mass balance condition for cobalt. Its total quantity equals: $[{\mathrm{Co}}_{\mathrm{Co}}^{•}]+[{\mathrm{Co}}_{\mathrm{Co}}^{\times }]+\left[{\mathrm{Co}}_{\mathrm{Co}}^{\prime }\right]=2-\left[{\mathrm{Fe}}_{\mathrm{Co}}^{\times }\right]=2-x$, i.e., it is lower than 2. The simplified model 1 was fitted to the *p*O_2_–*T*–δ-datasets of all the PBCF. The results are summarized in Table 1, and the fitted model surfaces are shown in Figure S9.

As seen from Figure S9 and Table 1, model 1 describes the measured *p*O_2_–*T*–δ-datasets relatively well. The coefficient of determination, *R*^2^, is sufficiently close to 1. The enthalpy of Co^3+^ disproportionation, $\Delta H_{3}^{○}$, was found to be constant, almost independent of the iron doping level and quite close to that of the undoped double cobaltites *RE*BaCo_2_O_6−δ_ [41]. The enthalpy of oxygen exchange reaction, $\Delta H_{1}^{○}$, somewhat grows with the concentration of iron in Co-sublattice. Interestingly, the enthalpy of quasichemical reaction (2) (Table 1), which describes the preferential localization of the oxygen vacancies in the rare-earth-element layers, shows the most significant variation with the iron content. It changes from a strongly negative value in the undoped PBC to zero in the PBCF6, indicating decreasing energy gain from such localization of oxygen vacancies within the Pr-layers. In this respect, the defect chemistry of the PBCF6 sample (*x* = 0.6) is very similar to that of LaBaCo_2_O_6−δ_ [41], i.e., here iron doping has the same effect on the defect structure as the increase in the radius of the A-site cation. Indeed, as discussed above (Section 3.1), PBCF samples with high doping level were occasionally obtained as cubic ‘simple’ perovskites [7,40], depending on the sample’s preparation history. The same is true for LaBaCo_2_O_6−δ_, which can also be synthesized as a cubic or a double perovskite [47]. Therefore, model 1 is in good general agreement with various structural, spectroscopic and thermodynamic experimental evidence.

**Figure 4:**
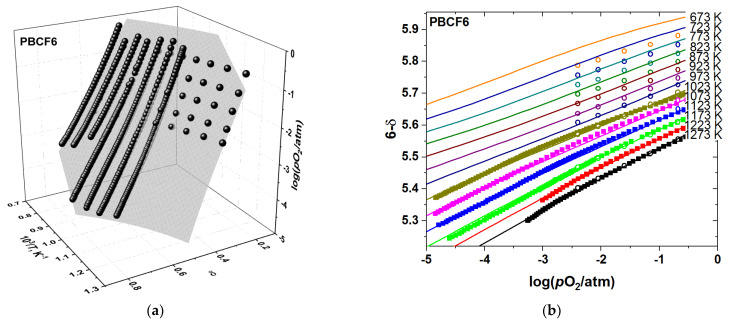
The results of fitting model 1 to the experimental *p*O_2_–*T*–δ-dataset of PBCF6: (**a**) 3D-fit; (**b**) 2D representation. The empty circles correspond to the TG results, the filled squares—the results obtained by CT. The surface in (**a**) and the lines in (**b**) represent the model 1 calculations.

However, observing closely the agreement between the model and the data in Figure S9 and considering the *R*^2^ decreasing with *x*, as seen in Table 1, reveals the decreasing goodness of fit with increasing concentration of iron. This is especially true for the sample with the largest amount of dopant, PBCF6, as seen in Figure 4. In addition, contrary to the undoped PBC sample, in the iron-containing PBCF the agreement between the oxygen content measured experimentally and that calculated according to model 1 worsens significantly at lower temperatures. This indicates that some additional contribution to the defect chemical model should be identified and included in the defect structure model.

There are at least two possibilities which can be discussed in this respect. First, following the authors of [48,49] one can consider the so-called hole–hole interaction, since the contribution of the unknown process to the defect equilibria should be more prominent at lower temperatures and higher oxygen content, where model 1 ceases to describe accurately the nonstoichiometry data. In this *T* and δ region, the concentration of electron holes also increases. The hole–hole interaction hypothesis is, however, inconsistent with the apparent absence of such an interaction in the undoped PBC, judging by the good agreement between the calculated and measured nonstoichiometry dependences in the whole temperature range studied. The same, in principle, may be argued for any other types of defect–defect interactions.

The second possibility, which, potentially, may account for the observed disagreement between the model and the experiment, is an energetic inhomogeneity of the oxygen sublattice, i.e., the presence of nonequivalent, more or less strongly bonded, oxygen atoms. The origin of this nonequivalence may be related to the different coordination environments. Indeed, in the undoped PBC all the oxygen sites are coordinated by two Co ions, whereas in the Fe-doped samples three different coordination environments are possible: (1) Co–O–Co, (2) Fe–O–Fe, and (3) Fe–O–Co. This suggests that, in principle, three distinct oxidation enthalpies may be identified. However, to make the corresponding defect chemical model simpler and avoid introducing too many fitting variables, we chose to consider two different oxidation enthalpies as the simplest possible scenario. The first one corresponds to the oxygen site coordinated by iron only, Fe–O–Fe, and the second one is characteristic of all other oxygen sites, both those with only cobalt and mixed, iron-cobalt, coordination. Therefore, to modify the defect structure model, two nonequivalent oxygen positions were introduced. Their concentrations were calculated assuming the statistical distribution of iron and cobalt in the B-sublattice of the double perovskite. Then, the following quasichemical reaction were proposed:

(9) $$O_{O1}^{\times }+2{\mathrm{Co}}_{\mathrm{Co}}^{•}+{\mathrm{Pr}}_{\mathrm{Pr}}^{\times }=\frac{1}{2}O_{2}+2{\mathrm{Co}}_{\mathrm{Co}}^{\times }+{\left({\mathrm{Pr}}_{\mathrm{Pr}}^{\times }-V_{O1}^{••}\right)}^{••}$$

(10) $$O_{O1}^{\times }+V_{O2}^{••}=O_{O2}^{\times }+V_{O1}^{••}$$

(11) $$2{\mathrm{Co}}_{\mathrm{Co}}^{\times }={\mathrm{Co}}_{\mathrm{Co}}^{•}+{\mathrm{Co}}_{\mathrm{Co}}^{\prime }$$

(12) $${\mathrm{Pr}}_{\mathrm{Pr}}^{\times }+V_{O1}^{••}={\left({\mathrm{Pr}}_{\mathrm{Pr}}^{\times }-V_{O1}^{••}\right)}^{••}$$

(13) $${\mathrm{Pr}}_{\mathrm{Pr}}^{\times }+V_{O2}^{••}={\left({\mathrm{Pr}}_{\mathrm{Pr}}^{\times }-V_{O2}^{••}\right)}^{••}$$

where $O_{O1}^{\times }$, $V_{O1}^{••}$, $O_{O2}^{\times }$ and $V_{O2}^{••}$ are the oxygen sites and the vacancies coordinated by either cobalt only or cobalt and iron, and the oxygen sites and the vacancies coordinated by iron only, respectively.

Since reactions (12) and (13) describe the preferential arrangement of oxygen ions in the rare-earth layers, in principle, it is possible to express them using the real crystallographic positions of various oxygen sites, as it was done in [50] for the defect structure model of Sr_3_Fe_2−*x*_Mo*_x_*O_7−δ_. However, since the ratio of rare-earth sites to oxygen sites in the rare-earth layers of PBCF is exactly 1:1, we found that it is easier to represent the vacancy ordering by the pseudocluster formation.

**Figure 5:**
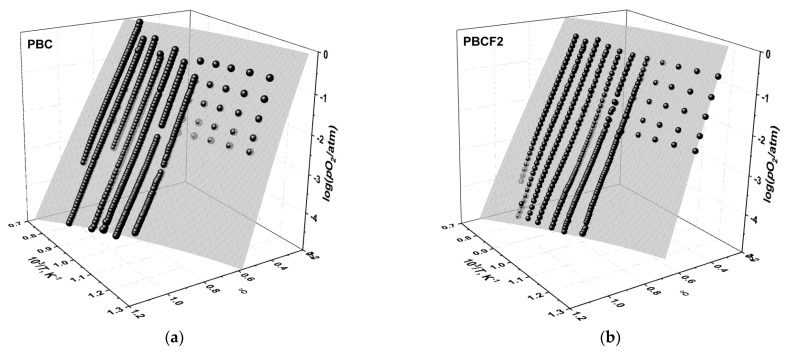
The results of fitting model 2 to the experimental *p*O_2_–*T*–δ-datasets of (**a**) PBC and (**b**) PBCF2. The surfaces and the filled spheres represent the model calculations and the experimental data, respectively.

**Figure 6:**
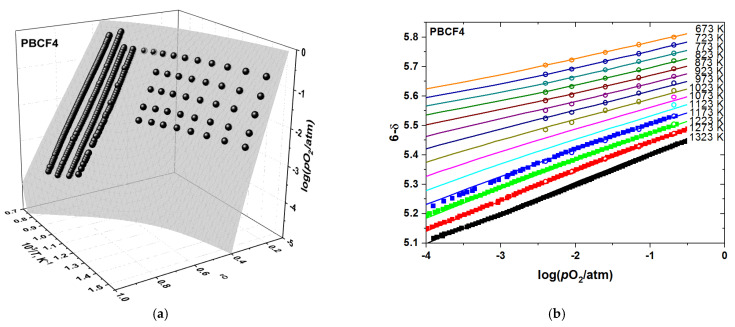
The results of fitting model 2 to the experimental *p*O_2_–*T*–δ-dataset of PBCF4: (**a**) 3D-fit; (**b**) 2D representation. The empty circles in (**b**) correspond to the TG results, the filled squares in (**b**)—to the results obtained by CT, and the spheres in a—to both the TG and CT nonstoichiometry measurement results. The surface in (**a**) and the lines in (**b**) represent the model 2 calculations.

**Figure 7:**
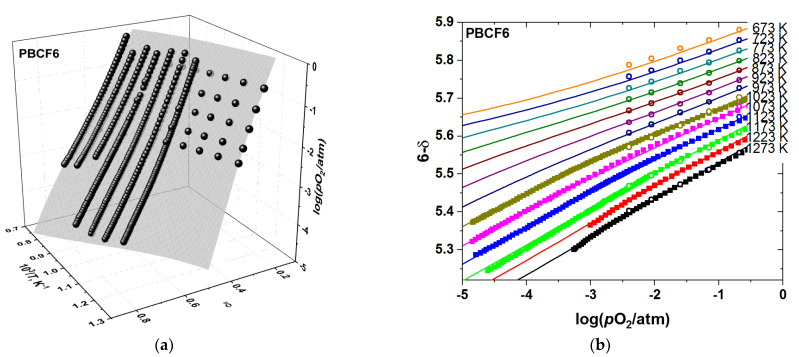
The results of fitting model 2 to the experimental *p*O_2_–*T*–δ-dataset of PBCF6: (**a**) 3D-fit; (**b**) 2D representation. The empty circles in (**b**) correspond to the TG results, the filled squares (in *b*)—to the results obtained by CT, and the spheres in a—to both the TG and CT nonstoichiometry measurement results. The surface in (**a**) and the lines in (**b**) represent the model 2 calculations.

**Figure 8:**
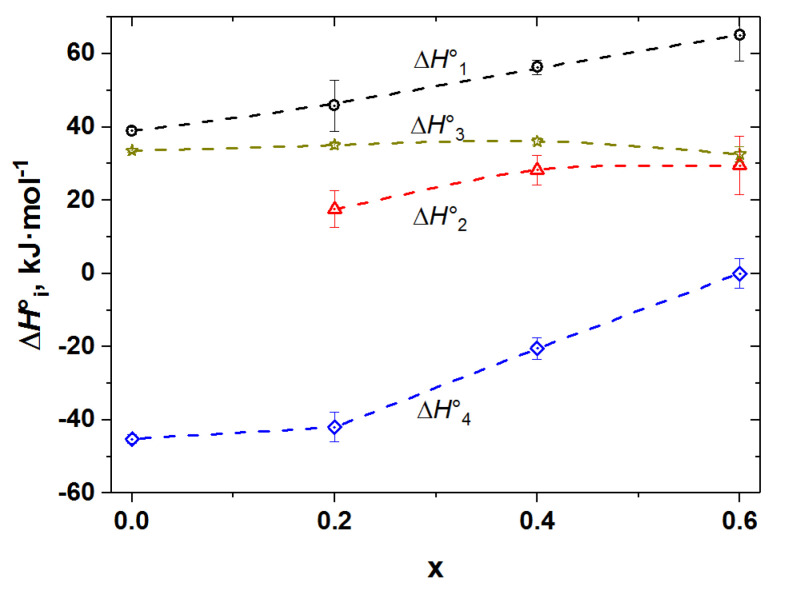
The standard enthalpies of quasichemical reactions (1)–(4) (see Table 2) vs. the concentration of dopant in PBCF.

The equilibrium constants of reactions (9)–(13) and the conditions of mass and charge balance result in the set of equations:

(14) K1=CoCo×2[PrPr×−VO1••••]pO212OO1×CoCo•2[PrPr×]=expΔS1○R−ΔH1○RTK2=OO2×VO1••OO1×VO2••=expΔS2○R−ΔH2○RTK3=CoCo•CoCo′[CoCo×]2=expΔS3○R−ΔH3○RTK4=PrPr×−VO1••••PrPr×VO1••=expΔS4○R−ΔH4○RTK5=PrPr×−VO2••••PrPr×VO2••=expΔS5○R−ΔH5○RTCoCo•+CoCo′+CoCo×=2−xFeCo×=xBaPr′=1PrPr×=1−PrPr×−VO1••••−PrPr×−VO2••••OO1×=61−α−VO1••−PrPr×−VO1••••OO2×=6α−VO2••−PrPr×−VO2••••PrPr×−VO1••••+VO1••+PrPr×−VO2••••+VO2••=δCoCo•+2VO1••+2PrPr×−VO1••••+2VO2••+2PrPr×−VO2••••=BaPr′+CoCo′

where α denotes the fraction of oxygen sites coordinated by iron only. Quasichemical reactions (12) and (13) were assumed, for the sake of simplicity, to possess the same thermodynamic parameters ($\Delta S_{4}^{○}=\Delta S_{5}^{○},\;\Delta H_{4}^{○}=\Delta H_{5}^{○}$). The set of equations (14), despite its apparent complexity, can be solved symbolically. Although the resulting equations are rather long and for this reason are not given here, the concentrations of all the defect species were obtained as along with the model 2 function *p*O_2_ = *f*′(*T*, δ, $\Delta H_{i}^{○}$, $\Delta S_{i}^{○}$) which was fitted to the experimental *p*O_2_–*T*–δ-datasets of PBCF. The results are summarized in Figure 5, Figure 6, Figure 7 and Figure 8 and in Table 2.

A comparison of Table 1 and Table 2 shows that the thermodynamic parameters of cobalt disproportionation (equations (3) within the tables) and localization of oxygen vacancies in Pr-layers (Equations (2) and (4)–(5) in Table 1 and Table 2, respectively) were found to be almost the same within the two models considered. Hence, the qualitative conclusions about the cobalt disproportionation and vacancies’ interaction with the rare-earth ions are the same irrespective of the model employed. Further, the enthalpy of oxygen exchange reaction (1) in model 2 (see Table 2) which involves $O_{O1}^{\times }$-sites, i.e., those that do not have two iron ions as their nearest neighbors, is also similar to the one estimated within the model 1 (see Table 1, reaction (1)) both in terms of its values and its tendency towards the gradual increase with the amount of the dopant, as seen in Figure 8. As for the oxygen exchange involving $O_{O2}^{\times }$-sites (having only iron as their nearest neighbors)—reaction (6) in Table 2—the value of its enthalpy (see Table 2) is the same for all the PBCF, being almost two times lower than that of reaction (1) (see Table 2) and somewhat higher than the one found for PrBaFe_2_O_5+*w*_ by Karen [51]. This is quite consistent with dominating oxidation state +3 of Fe in PBCFs as discussed above.

To sum up, model 2, which is a gradual improvement over model 1, is consistent with the available experimental structural, spectroscopic and thermodynamic data, and will be employed further for the analysis of oxygen transport in PBCF double perovskites.

### 3.3. Oxygen Diffusion and Surface Exchange

The chemical surface exchange coefficient, *k*_chem_, and the chemical diffusion coefficient, *D*_chem_, obtained as a result of ECR measurements for PBCFs double perovskites are summarized in Figures S10–S13 (see Supplementary). As seen, both *k*_chem_ and *D*_chem_ increase with temperature. *D*_chem_ of all the studied PBCF is almost independent of *p*O_2_, whereas *k*_chem_ of PrBaCo_2_O_6−δ_ is roughly proportional to $p_{O_{2}}^{0.5}$ at low *p*O_2_ and tends to saturate at higher *p*O_2_. This transitory behavior is more pronounced at high temperatures. For the Fe-doped samples, *k*_chem_ shows $\sim p_{O_{2}}^{0.5}$ dependence within the whole range of studied oxygen partial pressures.

**Figure 9:**
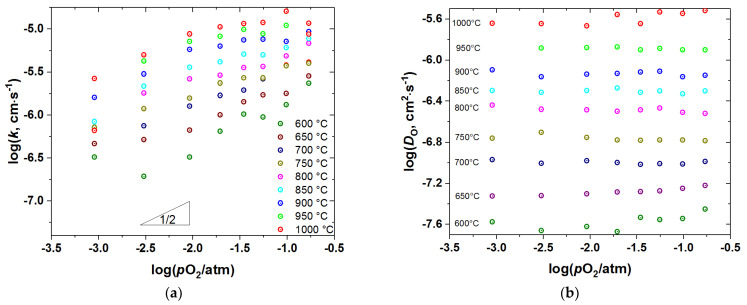
The surface exchange coefficient (**a**) and the self-diffusion coefficient (**b**) of PBC vs. *p*O_2_ at different temperatures.

**Figure 10:**
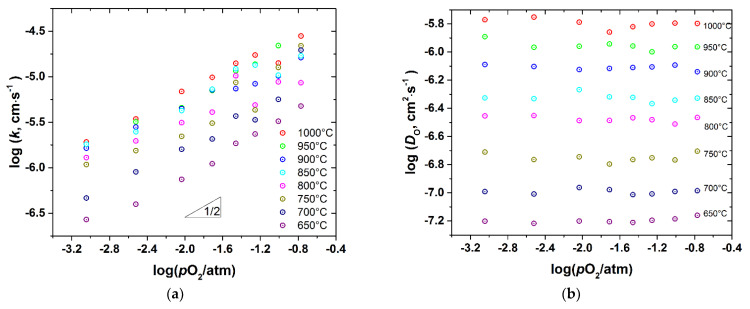
The surface exchange coefficient (**a**) and the self-diffusion coefficient (**b**) of PBCF2 vs. *p*O_2_ at different temperatures.

**Figure 11:**
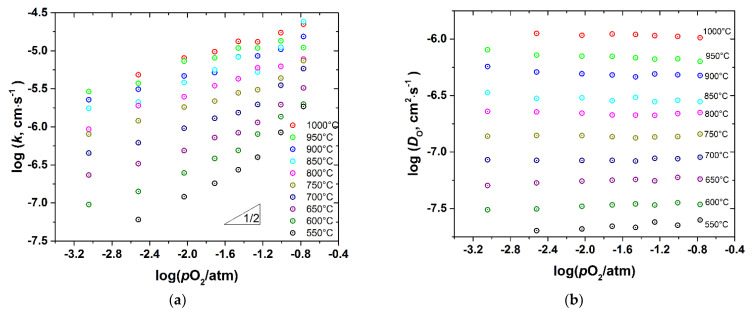
The surface exchange coefficient (**a**) and the self-diffusion coefficient (**b**) of PBCF4 vs. *p*O_2_ at different temperatures.

**Figure 12:**
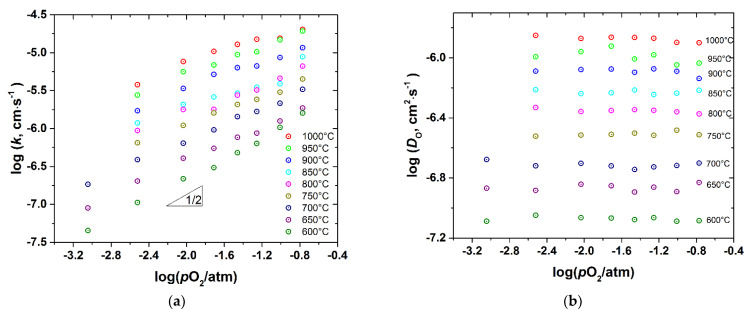
The surface exchange coefficient (**a**) and the self-diffusion coefficient (**b**) of PBCF6 vs. *p*O_2_ at different temperatures.

*D*_chem_ and *k*_chem_ were recalculated to the oxygen self-diffusion coefficient, *D*_O_, and the surface exchange coefficient, *k*, as described in the Experimental. The as-calculated values are presented in Figure 9, Figure 10, Figure 11 and Figure 12 as a function of *T* and *p*O_2_.

As seen, the surface exchange coefficient is thermally activated and shows *p*O_2_ dependence similar to *k*_chem_, i.e., for the undoped cobaltite *k*~$p_{O_{2}}^{0.5}$ at low *p*O_2_ and saturates at high *p*O_2_, whereas for the Fe-doped samples $k\sim p_{O_{2}}^{0.5}$ in the whole range of *p*O_2_. Similar, proportional to $p_{O_{2}}^{0.5}$, dependence of *k* was reported for PBC by Yoo et al. [9], who also found the change in the rate determining step of the oxygen exchange from the oxygen dissociative adsorption to the incorporation of oxygen adatoms upon *p*O_2_ increase from 0.1 to 0.95 atm. Later, similar conclusions were reported for *RE*BaCo_2_O_6–δ_ (*RE* = Pr, Sm, Gd) [10]. This explanation is consistent with the transitory behavior of *k* observed in the present work. Indeed, Yoo et al. [9] found the rate of incorporation of oxygen adatoms to be almost independent of *p*O_2_, contrary to the oxygen dissociative adsorption which is roughly proportional to $p_{O_{2}}^{0.8}$.

In turn, the oxygen self-diffusion coefficient in PBCF, as shown in Figure 9, Figure 10, Figure 11 and Figure 12, depends mainly on temperature and is practically insensitive to variations of *p*O_2_. Similar observations were reported in [10] for the undoped double perovskite cobaltites *RE*BaCo_2_O_6−δ_ (*RE* = Pr, Sm, Gd). The fact that *D*_O_ in double perovskites was found to be independent of *p*O_2_ can be explained from the viewpoint of the oxygen diffusion mechanism in these oxides. As revealed by a number of studies [6,14,15,16,17,18,19,20,21,22] using different techniques, oxygen ions diffuse in *RE*BaCo_2_O_6−δ_ along the CoO_2−_planes through the oxygen vacancies coming from the rare-earth-element planes. These planes, therefore, serve only as a source-sink for the oxygen vacancies and do not participate in the oxygen transfer directly. As a consequence, the amount of oxygen vacancies in the Co(Fe)O_2−_planes should limit the diffusion of oxygen. In the defect chemistry modelling discussed above, $V_{O1}^{••}$ and $V_{O2}^{••}$ represent such diffusion-active vacancies in PBCF. The calculations according to model 2 show that, for example, at 1000 °C the *p*O_2_ increase from 10^−3^ to 0.21 atm, i.e., by almost three orders of magnitude, leads to the increase in the total concentration of such vacancies in the undoped PBC sample by 2.86 times only, or by ~0.46 in the logarithmic scale. In the iron-doped samples, this increase is even lower: for example, in PBCF6 under similar conditions the total concentration of the oxygen vacancies participating in the diffusion increases by 1.433 times, or by ~0.16 in the logarithmic scale. Such changes are well within the uncertainties of the diffusion coefficients determined by fitting the conductivity relaxation curves, which causes the observed *D*_O_ values to appear independent of *p*O_2_.

**Figure 13:**
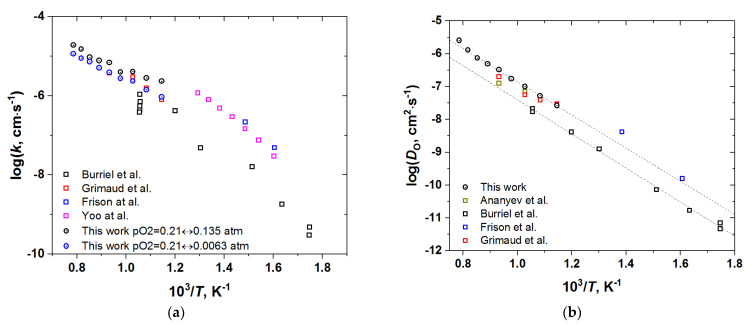
The surface exchange coefficient (**a**) and the oxygen self-diffusion coefficient (**b**) of PBC vs. temperature in comparison with the available literature data [6,7,8,9,10]. The dashed lines in (**b**) correspond to Arrhenius-type dependences with the activation energy *E*_A_ = 1.02 eV, as obtained in [6], and (1.00 ± 0.04) eV, estimated in this work.

It is also of interest to compare the temperature dependencies of the oxygen self-diffusion coefficient and the surface exchange coefficient for the undoped PBC sample with the data available in the literature. Such a comparison is shown in Figure 13. Please note that since *D*_O_ did not show the *p*O_2_ dependence, its values, for the sake of comparison, were averaged at each *T* over the whole *p*O_2_ range studied. For *k*, in turn, either the values at *p*O_2_ = 0.21 atm or those averaged in the range of 0.0063–0.21 atm were plotted in Figure 13, since these *p*O_2_ correspond to the oxygen pressures most commonly employed in the literature for isotope exchange or conductivity relaxation, respectively. A very good agreement between various datasets presented in Figure 13 is obvious, irrespective of the particular experimental technique employed. This is an additional confirmation of the validity of the results obtained in this work.

**Figure 14:**
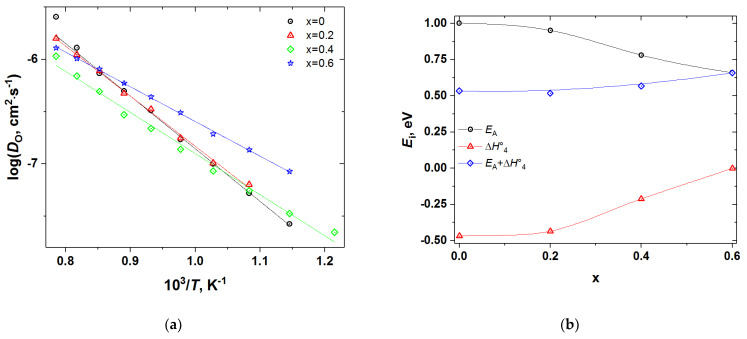
(**a**) The oxygen self-diffusion coefficient of PBCF double perovskites vs. *T*; (**b**) The activation energy of oxygen self-diffusion in comparison with $\Delta H_{4}^{○}$—the enthalpy gain of oxygen vacancies’ localization in the Pr-layers (reactions (4)–(5) in Table 2).

A comparison of Figure 9a, Figure 10a, Figure 11a, Figure 12a shows that iron doping almost does not influence the value of *k*, since all the PBCFs studied were shown to possess comparable surface exchange coefficients within the investigated ranges of *T* and *p*O_2_. Grimaud et al. [7] also showed that samples of PBC and PrBaCo_1.5_Fe_0.5_O_6−δ_ (PBCF5) possess almost the same surface exchange coefficient. At the same time, as clearly seen in Figure 14, the activation energy of oxygen self-diffusion decreases with *x*. As a result, as seen in Figure 14a, all the iron-doped PBCF samples possess *D*_O_ either comparable with or higher than the *D*_O_ of PBC in the range of intermediate temperatures (*T* < 600–700 °C). This is especially pronounced for the PBCF6 sample, which demonstrates the lowest activation energy of oxygen self-diffusion and the largest *D*_O_ already at *T* < 900 °C and, for example, at 600 °C its *D*_O_ is more than three times that of undoped PBC.

These observations are fully in line with both the data on oxygen permeability through PBCF membranes [4] and those on polarization resistance of PBCF cathodes [40]. In both cases, it was shown that in the intermediate temperature range the iron-doped samples with *x* = 0.4–0.6 have either similar or better performances compared to the undoped cobaltite PBC. However, Grimaud et al. [7] came to the opposite conclusion that polarization resistance increases with iron content. It seems the discrepancy between the works [7] and [40] may be related to the electrolyte employed for the study. Zou et al. [40] used substituted ceria, whereas Grimaud et al. [7]—proton-conducting BaCe_0.9_Y_0.1_O_3-δ_. The latter study reported negative influence of water vapor on the polarization resistance of iron-doped samples contrary to the undoped cobaltite. Since with a proton-conducting electrolyte it is almost impossible to avoid the water vapor influence, this may be the source of the disagreement between the results of [7] and [40]. However, this is just one possibility among many, since electrode performance depends on a lot of factors, including the morphology. Therefore, it is always difficult to compare electrochemical characteristics measured in different works.

At the same time, Grimaud et al. [7] also observed decreased activation energy of oxygen self-diffusion in PBCF5 as compared to PBC, which is in agreement with our results (see Figure 14). The origin of the fact that Fe doping causes the decrease in the oxygen self-diffusion activation energy in PBCF can be understood based on the diffusion mechanism discussed above and results of the defect structure modelling. Indeed, since oxygen transfer in the CoO_2−_planes requires the presence of oxygen vacancies ($V_{O1}^{••}$ and $V_{O2}^{••}$ in the defect chemical model 2) the source of which are the PrO_1−δ_-layers where vacancies are trapped in the form of quasi-complexes ${\left({\mathrm{Pr}}_{\mathrm{Pr}}^{\times }-V_{O1}^{••}\right)}^{••}$ and ${\left({\mathrm{Pr}}_{\mathrm{Pr}}^{\times }-V_{O2}^{••}\right)}^{••}$, the dissociation enthalpy of these complexes must contribute to the activation energy of self-diffusion in double perovskites. Therefore, the higher (more positive) the enthalpy of vacancy trapping in Pr-layers ($\Delta H_{4}^{○}$ in the model 2) the lower should be the activation energy of oxygen self-diffusion. This type of inverse correlation between *E*_A_ and $\Delta H_{4}^{○}$ can be observed in Figure 14b and in Figure S14. Therefore, activation energy may be represented by the sum of, at least, two contributions: the formation enthalpy of oxygen vacancies in CoO_2−_layers, equal to $-\Delta H_{4}^{○}$ (see Table 2), and the migration enthalpy of these vacancies in the same layers, Δ*H*_m_:

(15) $$E_{A}=-\Delta H_{4}^{○}+\Delta H_{m}.$$

Hence, the Δ*H*_m_ term can be estimated as the sum $E_{A}+\Delta H_{4}^{○}$. The as-estimated Δ*H*_m_ is shown in Figure 14b as a function of iron content *x* in PBCF. As seen, Δ*H*_m_ gradually grows from about 0.53 eV for undoped PBC to 0.66 eV for PBCF6 sample, reflecting the increasing difficulty of the vacancy migration with the increase in the dopant concentration. Therefore, the main positive implication of the Fe doping for oxygen diffusion in double perovskite cobaltites is that it seems to facilitate the formation of the mobile oxygen vacancies (i.e., those in the CoO_2−_layers). However, this happens at the cost of somewhat increase in their migration enthalpy.

## 4. Conclusions

Oxygen content in the Fe-doped double perovskites PBCF was measured as a function of *T* and *p*O_2_. The as-obtained *p*O_2_–*T*–δ-datasets were successfully described using the defect chemical model based on the reactions of oxygen exchange with the surrounding atmosphere, Co^3+^ disproportionation and vacancies’ preferential localization in the Pr-layers. The different coordination environment of oxygen in the Fe-doped samples was also taken into account in the model, since this was shown to be essential for the accurate description of the behavior of oxygen content in the PBCF, especially at lower temperatures. Fe-doping was also shown to significantly affect the enthalpy gain of oxygen vacancies’ localization in the Pr-layers, decreasing the energetic cost of the diffusion-active oxygen vacancy formation in the CoO_2−_layers. This leads to the decreasing activation energy of oxygen self-diffusion with increasing concentration of iron in PBCF, as verified by the results of electrical conductivity relaxation measurements. The linear correlation of the activation energy of oxygen self-diffusion with the enthalpy of formation of oxygen vacancies in CoO_2−_layers also provides an independent confirmation of the oxygen self-diffusion mechanism in the layered double perovskite cobaltites, studied earlier by neutron diffraction [15,19,22] and computations [14,16,17,18,20,21].

We found that iron doping almost does not affect either the mechanism of surface exchange or the value of the surface exchange coefficient. The results of the present study are quite consistent with the rate-determining step of the oxygen exchange in the Fe-doped samples being the oxygen dissociative adsorption in the whole *p*O_2_ and *T* ranges investigated. At the same time, in the undoped PBC cobaltite at high temperatures, the rate-determining step changes from the O_2_ dissociative adsorption to the incorporation of oxygen adatoms upon *p*O_2_ increase.

In addition to retaining the already high values of the surface exchange coefficient, typical of the undoped PBC oxide, iron doping decreases the activation energy of oxygen self-diffusion and promotes the oxygen self-diffusion in PBCF in the intermediate temperature range. These findings indicate that iron doping enhances the oxygen transport properties of double perovskite membrane materials. Furthermore, the previous studies demonstrated that iron doping increases the relative thermodynamic stability of double perovskites [31], which is also beneficial for their practical applications.

The decrease in the activation energy of oxygen self-diffusion with iron concentration in PBCF, demonstrated in this work, and its interpretation through the defect structure analysis, possess twofold significance. On the one hand, they provide an important link between the particularities of the defect chemistry of double perovskites and their transport properties. On the other hand, as shown in this work, the defect chemistry guidance allows one to optimize the properties of double perovskite materials through the targeted variation of their defect energetics. This can be regarded as one of the general, not limited to double perovskites, methods for the intentional modification and development of novel materials for various practical applications, including membranes and energy conversion devices.

## Figures and Tables

**Table 1:** Results of fitting model 1 to the measured *p*O_2_–*T*–δ-datasets of PBCFs.

No.	Reaction	Sample *							
		PBC		PBCF2		PBCF4		PBCF6	
		$\Delta H_{i}^{○}$	$\Delta S_{i}^{○}$	$\Delta H_{i}^{○}$	$\Delta S_{i}^{○}$	$\Delta H_{i}^{○}$	$\Delta S_{i}^{○}$	$\Delta H_{i}^{○}$	$\Delta S_{i}^{○}$
1.	$O_{O}^{\times }+2{\mathrm{Co}}_{\mathrm{Co}}^{•}+{\mathrm{Pr}}_{\mathrm{Pr}}^{\times }=\frac{1}{2}O_{2}+{\left(V_{O}^{••}-{\mathrm{Pr}}_{\mathrm{Pr}}^{\times }\right)}^{••}+2{\mathrm{Co}}_{\mathrm{Co}}^{\times }$	39 ±1	45 ±1	50 ±2	53 ±3	51 ±1	48 ±1	61 ±5	36 ±6
2.	${\mathrm{Pr}}_{\mathrm{Pr}}^{\times }+V_{O}^{••}={\left(V_{O}^{••}-{\mathrm{Pr}}_{\mathrm{Pr}}^{\times }\right)}^{••}$	−45 ±1	0	−42 ±4	0	−18 ±5	0	0	0
3.	$2{\mathrm{Co}}_{\mathrm{Co}}^{\times }={\mathrm{Co}}_{\mathrm{Co}}^{\prime }+{\mathrm{Co}}_{\mathrm{Co}}^{•}$	33.6 ±0.4	0	36 ±1	0	37 ±1	0	34 ±3	0
	*R* ^2^	0.995		0.995		0.985		0.975	

* The numbers following ‘±’ represent the expanded uncertainty (~95% confidence level).

**Table 2:** Results of the fitting model 2 to the measured *p*O_2_–*T*–δ-datasets of PBCFs.

No.	Reaction	Sample *							
		PBC		PBCF2		PBCF4		PBCF6	
		$\Delta H_{i}^{○}$	$\Delta S_{i}^{○}$	$\Delta H_{i}^{○}$	$\Delta S_{i}^{○}$	$\Delta H_{i}^{○}$	$\Delta S_{i}^{○}$	$\Delta H_{i}^{○}$	$\Delta S_{i}^{○}$
1.	$O_{O1}^{\times }+2{\mathrm{Co}}_{\mathrm{Co}}^{•}+{\mathrm{Pr}}_{\mathrm{Pr}}^{\times }=\frac{1}{2}O_{2}+{\left(V_{O1}^{••}-{\mathrm{Pr}}_{\mathrm{Pr}}^{\times }\right)}^{••}+2{\mathrm{Co}}_{\mathrm{Co}}^{\times }$	39 ±1	45 ±1	46 ±7	52 ±3	56 ±2	53 ±1	65 ±7	45 ±2
2.	$O_{O1}^{\times }+V_{O2}^{••}=O_{O2}^{\times }+V_{O1}^{••}$	-	-	18 ±5	0 **	28 ±4	0 **	30 ±8	0 **
3.	$2{\mathrm{Co}}_{\mathrm{Co}}^{\times }={\mathrm{Co}}_{\mathrm{Co}}^{\prime }+{\mathrm{Co}}_{\mathrm{Co}}^{•}$	33.6 ±0.4	0 **	35 ±1	0 **	36 ±1	0 **	33 ±2	0 **
4.	${\mathrm{Pr}}_{\mathrm{Pr}}^{\times }+V_{O1}^{••}={\left({\mathrm{Pr}}_{\mathrm{Pr}}^{\times }-V_{O1}^{••}\right)}^{••}$	−45 ±1	0 **	−42 ±4	0 **	−20 ±3	0 **	0	0 **
5.	${\mathrm{Pr}}_{\mathrm{Pr}}^{\times }+V_{O2}^{••}={\left({\mathrm{Pr}}_{\mathrm{Pr}}^{\times }-V_{O2}^{••}\right)}^{••}$								
6.	$O_{O2}^{\times }+2{\mathrm{Co}}_{\mathrm{Co}}^{•}+{\mathrm{Pr}}_{\mathrm{Pr}}^{\times }=\frac{1}{2}O_{2}+{\left(V_{O2}^{••}-{\mathrm{Pr}}_{\mathrm{Pr}}^{\times }\right)}^{••}+2{\mathrm{Co}}_{\mathrm{Co}}^{\times }$ ***	-	-	28 ±10	52 ±3	28 ±6	53 ±1	35 ±11	45 ±2
	α ****	0		0.01		0.04		0.09	
	*R* ^2^	0.995		0.996		0.996		0.995	

* Numbers following ‘±’ represent the expanded uncertainty (~95% confidence level); ** assumed to equal 0 during the fitting procedure, for details see [41]; *** this is a combination of reactions (1), (2), (4) and (5), and its thermodynamic parameters were calculated after the fitting from the $\Delta H_{i}^{○}$, $\Delta S_{i}^{○}$ of those reactions; **** calculated using binomial distribution assuming statistical distribution of Co and Fe in the B-sublattice.

## Data Availability

The reported data are available by a reasonable request from the corresponding authors.

## Supplementary Materials

The following supporting information, which can be downloaded at https://www.mdpi.com/article/10.3390/membranes12121200/s1, contains: Figure S1. Typical results of the morphology and chemical composition study for PrBaCo_1.4_Fe_0.6_O_5.92_, representative for all PrBaCo_2−*x*_Fe_*x*_O_6−δ_ (PBCF) samples: (a) electron image in the backscattered electrons (BSE) mode; element distribution maps (energy-dispersive X-ray (EDX) spectroscopy): (b) cumulative Pr + Ba + Co + Fe; (c) Pr; (d) Ba; (e) Co; (f) Fe; Figure S2. Typical EDX spectra of the studied samples of PrBaCo_2−*x*_Fe_*x*_O_6−δ_ (PBCF): PrBaCo_2_O_5.77_ (PBC), PrBaCo_1.8_Fe_0.2_O_5.84_ (PBCF2), PrBaCo_1.6_Fe_0.4_O_5.87_ (PBCF4) and PrBaCo_1.4_Fe_0.6_O_5.92_ (PCBF6); Figure S3. The normalized total conductivity of PrBaCo_1.4_Fe_0.6_O_6−δ_ (PBCF6) at 900 °C during the various *p*O_2_ switches; Figure S4. The result of fitting of the solution of Fick’s 2nd law to the normalized total conductivity of PBCF6 at 900 °C during the log(*p*O_2_/atm) switching from −0.88 to −1.14; Table S1. Refined lattice parameters and oxygen content of the as-prepared PrBaCo_2−*x*_Fe_*x*_O_6−δ_ (PBCF); Figure S5. In situ X-ray diffraction (XRD) patterns of PBC: (a) log(*p*O_2_/atm) = −0.68; (b) log(*p*O_2_/atm) = −3; (c) zoomed view of some fragments of (b) close to phase transition *P*4*/mmm*↔*Pmmm*; Figure S6. In situ XRD patterns of PBCF2: (a) log(*p*O_2_/atm) = −0.68; (b) log(*p*O_2_/atm) = −3; Figure S7. In situ XRD patterns of PBCF4: (a) log(*p*O_2_/atm) = −0.68; (b) log(*p*O_2_/atm) = −3; Figure S8. In situ XRD patterns of PBCF6: (a) log(*p*O_2_/atm) = −0.68; (b) log(*p*O_2_/atm) = −3; Figure S9. The results of fitting model 1 to the experimental *p*O_2_–T–δ-datasets of (a) PBC; (b) PBCF2; (c) PBCF4; (d) PBCF6; Figure S10. The chemical surface exchange coefficient (a) and the chemical diffusion coefficient (b) of PBC vs. *p*O_2_; Figure S11. The chemical surface exchange coefficient (a) and the chemical diffusion coefficient (b) of PBCF2 vs. *p*O_2_; Figure S12. The chemical surface exchange coefficient (a) and the chemical diffusion coefficient (b) of PBCF4 vs. *p*O_2_; Figure S13. The chemical surface exchange coefficient (a) and the chemical diffusion coefficient (b) of PBCF6 vs. *p*O_2_; Figure S14. The activation energy of self-diffusion vs. the enthalpy of oxygen vacancies’ localization in the Pr-layers for the PBCF oxides with different iron content.
