# Supplementary material for: Nonstoichiometry, Defect Chemistry and Oxygen Transport in Fe-Doped Layered Double Perovskite Cobaltite PrBaCo2−xFexO6−δ (x = 0–0.6) Membrane Materials
Source: Membranes (Basel). 2022 Nov 28;12(12):1200. doi: 10.3390/membranes12121200 (PMC9783566; doi:10.3390/membranes12121200)
Supplement: Supplementary file 1 [file membranes-12-01200-s001.zip › membranes-2038876-supplementary.pdf]

membranes MDPI

Supplementary Materials

Nonstoichiometry, Defect Chemistry and Oxygen Transport in Fe-Doped Layered Double Perovskite Cobaltite $PrBaCo_{2-x}Fe_xO_{6-\delta}$ (x=0–0.6) Membrane Materials

Ivan L. Ivanov, Petr O. Zakiryanov, Vladimir V. Sereda, Maxim O. Mazurin, Dmitry A. Malyshkin, Andrey Yu. Zuev and Dmitry S. Tsvetkov *

Institute of Natural Sciences and Mathematics, Ural Federal University, 19 Mira St., 620002 Ekaterinburg, Russia

* Correspondence: dmitry.tsvetkov@urfu.ru

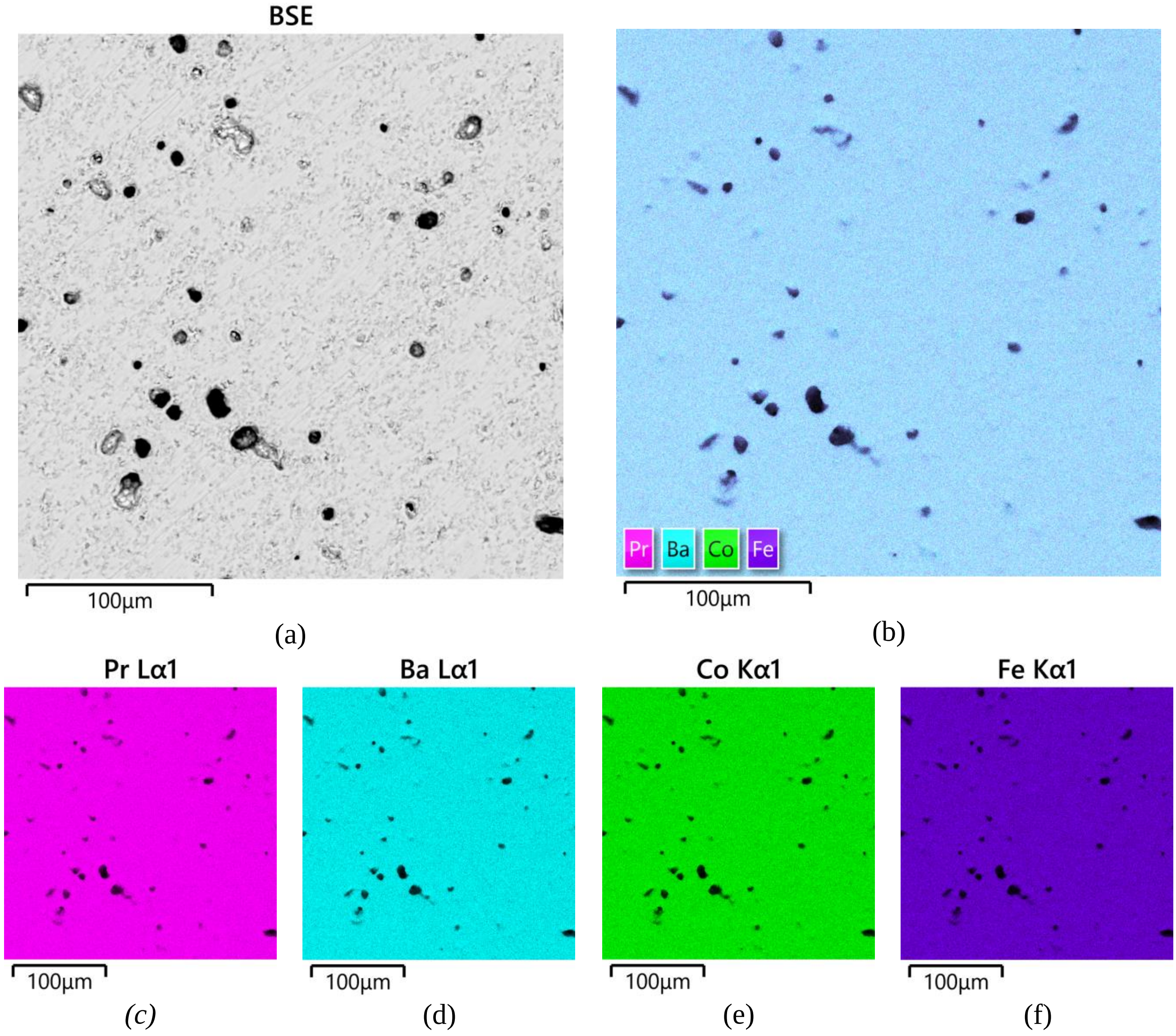

Figure S1. Typical results of the morphology and chemical composition study for $PrBaCo_{1.4}Fe_{0.6}O_{5.92}$, representative for all $PrBaCo_{2-x}Fe_xO_{6-\delta}$ (PBCF) samples: (**a**) electron image in the backscattered electrons (BSE) mode; element distribution maps (energy-dispersive X-ray (EDX) spectroscopy): (**b**) cumulative Pr+Ba+Co+Fe; (**c**) Pr; (**d**) Ba; (**e**) Co; (**f**) Fe.

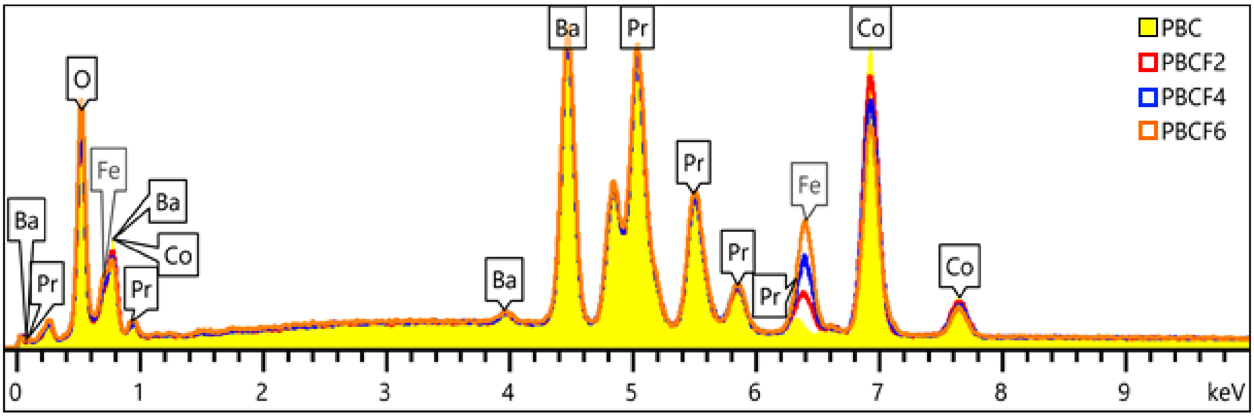

Figure S2. Typical EDX spectra of the studied samples of $PrBaCo_{2-x}Fe_xO_{6-\delta}$ (PBCF): $PrBaCo_2O_{5.77}$ (PBC), $PrBaCo_{1.8}Fe_{0.2}O_{5.84}$ (PBCF2), $PrBaCo_{1.6}Fe_{0.4}O_{5.87}$ (PBCF4) and $PrBaCo_{1.4}Fe_{0.6}O_{5.92}$ (PCBF6).

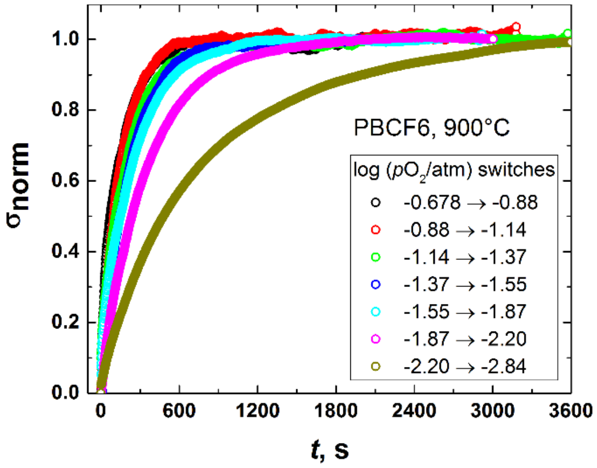

Figure S3. The normalized total conductivity of $PrBaCo_{1.4}Fe_{0.6}O_{6-\delta}$ (PBCF6) at 900 °C during the various pO_2 switches.

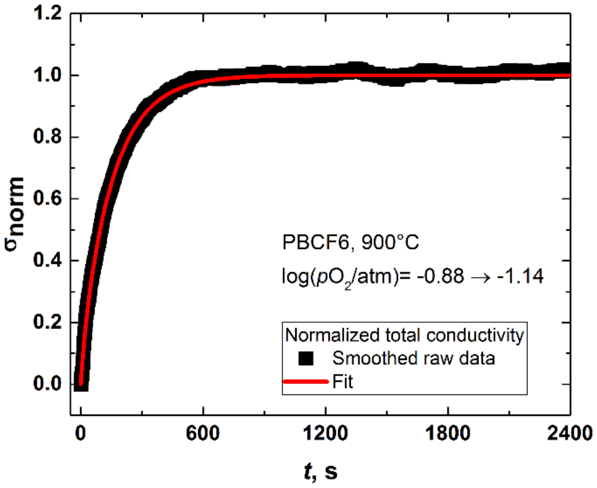

Figure S4. The result of fitting of the solution of Fick's 2nd law to the normalized total conductivity of PBCF6 at 900 °C during the log(pO_2/atm) switching from −0.87 to −1.13: experimental data – black squares, fitted function – red line.

Table S1. Refined lattice parameters, oxygen content and cation mass fractions in the as-prepared $PrBaCo_{2-x}Fe_xO_{6-\delta}$ (PBCF). $PrBaCo_2O_{6-\delta}$ (PBC), $PrBaCo_{1.8}Fe_{0.2}O_{6-\delta}$ (PBCF2), $PrBaCo_{1.6}Fe_{0.4}O_{6-\delta}$ (PBCF4) and $PrBaCo_{1.4}Fe_{0.6}O_{6-\delta}$ (PCBF6).

Sample	Space Group	Cell Parameters (Å)	Oxygen content	Chemical composition (wt.%)*	
				Meas.	Calc.
PBC	*P*4/*mmm*	*a* = 3.9039 ± 0.0001<br>*b* = 3.9039 ± 0.0001<br>*c* = 7.6311 ± 0.0001	5.77 ± 0.02	Pr–28.77<br>Ba–28.22<br>Co–24.03	Pr–28.85<br>Ba–28.12<br>Co–24.13
PBCF2	*P*4/*mmm*	*a* = 3.9029 ± 0.0009<br>*b* = 3.9029 ± 0.0009<br>*c* = 7.6444 ± 0.0002	5.84 ± 0.02	Pr-28.80<br>Ba–28.12<br>Co–21.50<br>Fe–2.33	Pr–28.82<br>Ba–28.09<br>Co–21.70<br>Fe–2.28
PBCF4	*P*4/*mmm*	*a* = 3.9010 ± 0.0001<br>*b* = 3.9010 ± 0.0001<br>*c* = 7.6581 ± 0.0003	5.87 ± 0.02	Pr–28.79<br>Ba–28.12<br>Co–19.31<br>Fe–4.60	Pr–28.83<br>Ba–28.10<br>Co–19.29<br>Fe–4.57
PBCF6	*P*4/*mmm*	*a* = 3.9083 ± 0.0001<br>*b* = 3.9083 ± 0.0001<br>*c* = 7.6776 ± 0.0003	5.92 ± 0.02	Pr–28.80<br>Ba–28.11<br>Co–16.82<br>Fe–6.80	Pr–28.82<br>Ba–28.09<br>Co–16.87<br>Fe–6.85

*Expanded uncertainty (95% confidence level) *U* =0.3 wt%.

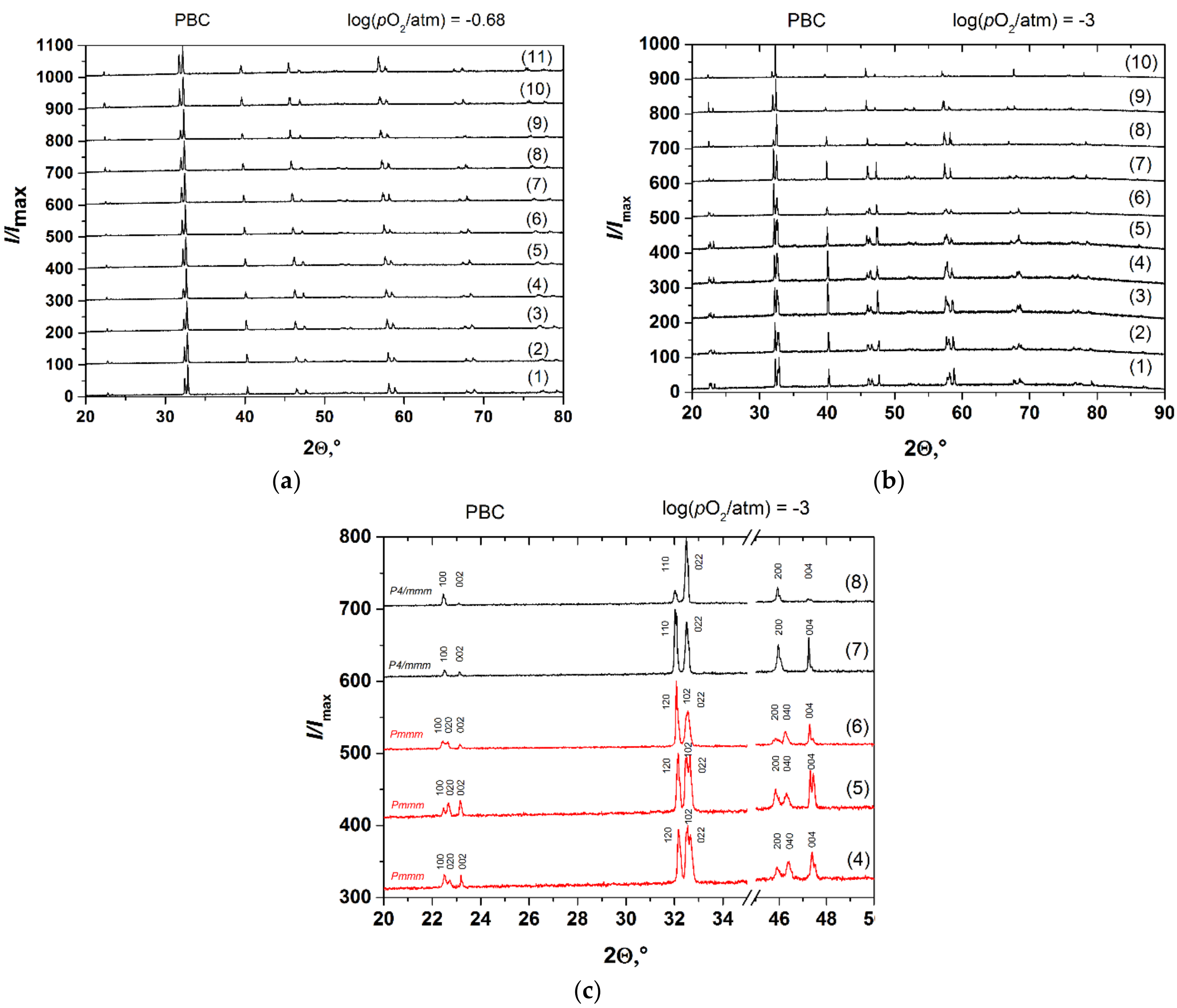

Figure S5. In situ X-ray diffraction (XRD) patterns of PBC: (**a**) log(pO_2/atm) = −0.68; (**b**) log(pO_2/atm) = −3; (**c**) zoomed view of some fragments of (b) close to phase transition *P*4/*mmm*↔*Pmmm*. 1—25 °C, 2—100 °C, 3—200 °C, 4—300 °C, 5—400 °C, 6—500 C, 7—600 C, 8—700 °C, 9—800 °C, 10—900 °C, 11—1000 °C.

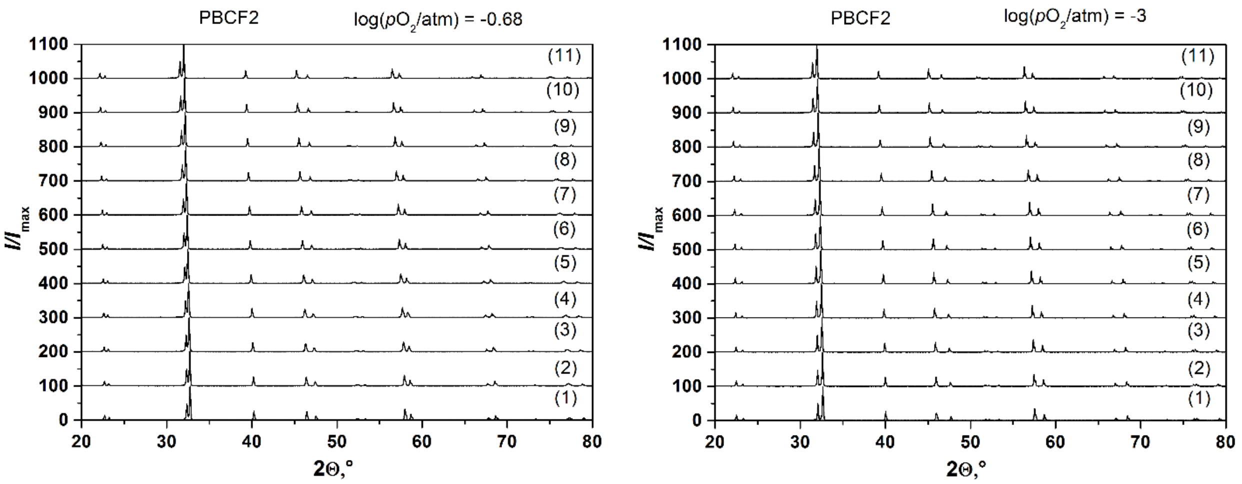

(**a**) (**b**)

Figure S6. In situ XRD patterns of PBCF2: (**a**) log(pO_2/atm) = −0.68; (**b**) log(pO_2/atm) = −3. 1—25 °C, 2—100 °C, 3—200 °C, 4—300 °C, 5—400 °C, 6—500 °C, 7—600 °C, 8—700 °C, 9—800 °C, 10—900 °C, 11—1000 °C.

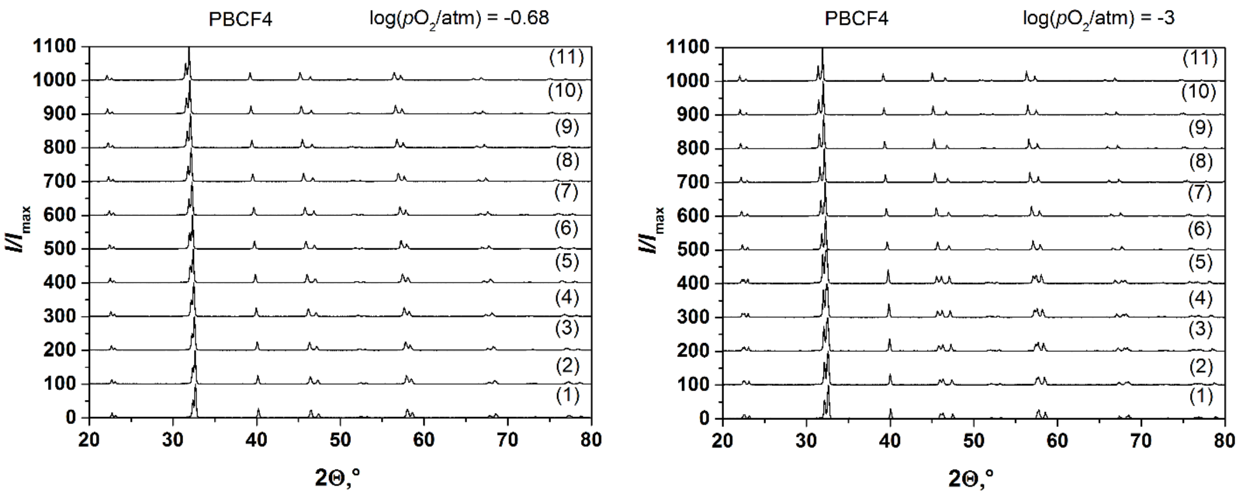

(**a**) (**b**)

Figure S7. In situ XRD patterns of PBCF4: (**a**) log(pO_2/atm)=-0.68; (**b**) log(pO_2/atm)=-3. 1—25 °C, 2—100 °C, 3—200 °C, 4—300 °C, 5—400 °C, 6—500 °C, 7—600 °C, 8—700 °C, 9—800 °C, 10—900 °C, 11—1000 °C.

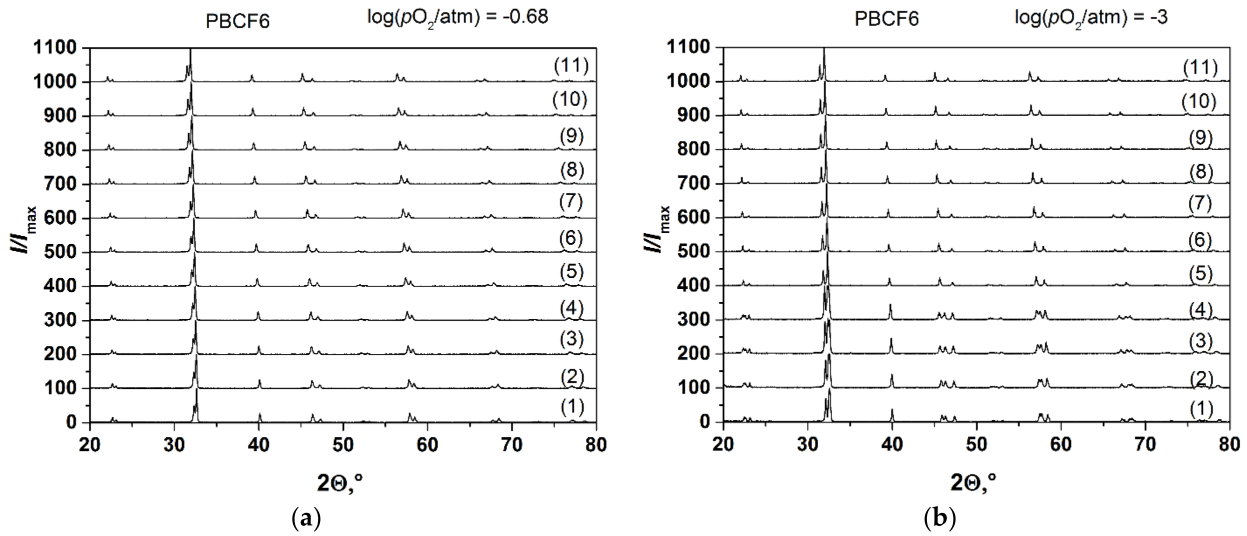

Figure S8. In situ XRD patterns of PBCF6: (**a**) log(pO_2/atm)=-0.68; (**b**) log(pO_2/atm)=-3. 1—25 °C, 2—100 °C, 3—200 °C, 4—300 °C, 5—400 °C, 6—500 °C, 7—600 °C, 8—700 °C, 9—800 °C, 10—900 °C, 11—1000 °C.

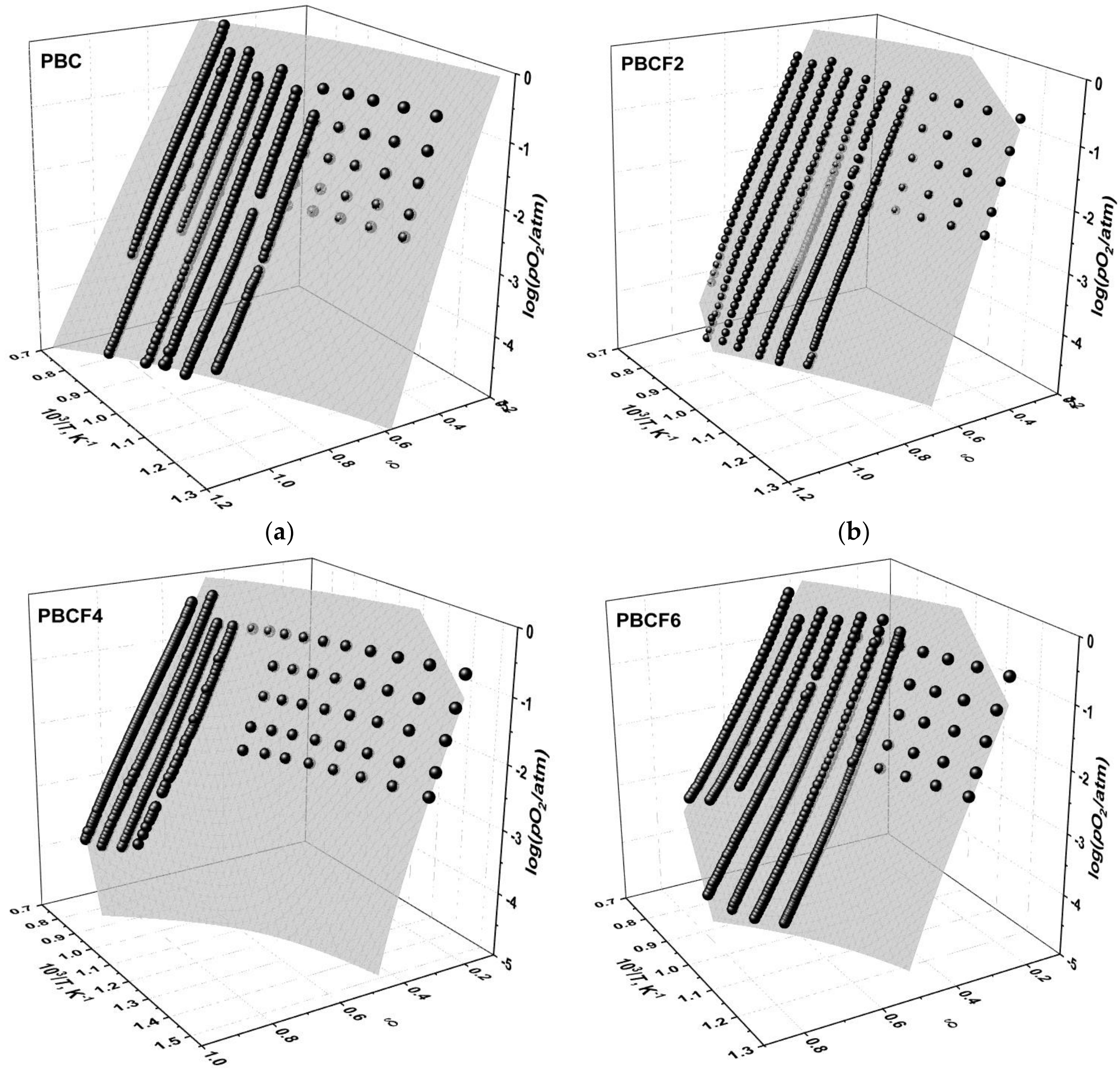

Figure S9. The results of fitting model 1 to the experimental pO_2–T–δ-datasets of (**a**) PBC; (**b**) PBCF2; (**c**) PBCF4; (**d**) PBCF6. The surfaces represent model calculations, the points—experimental data.

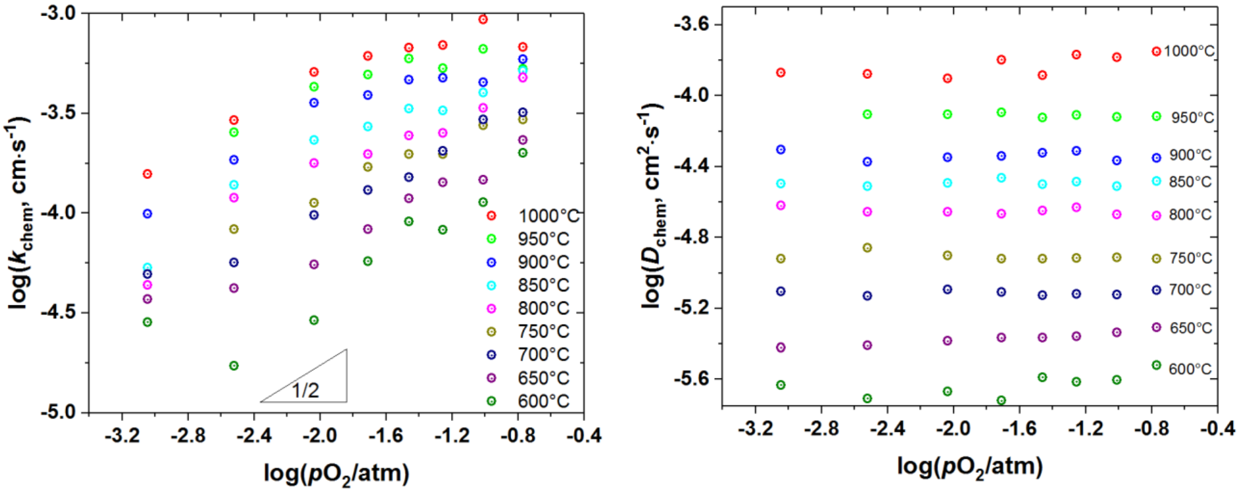

(**a**) (**b**)

Figure S10. The chemical surface exchange coefficient (**a**) and the chemical diffusion coefficient (**b**) of PBC vs. pO_2.

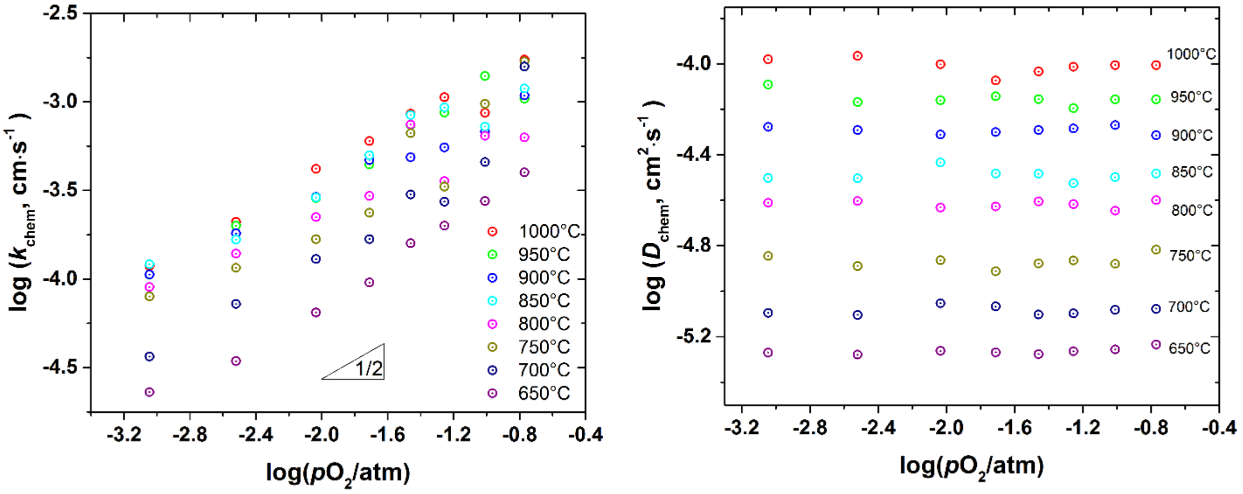

(**a**) (**b**)

Figure S11. The chemical surface exchange coefficient (**a**) and the chemical diffusion coefficient (**b**) of PBCF2 vs. pO_2.

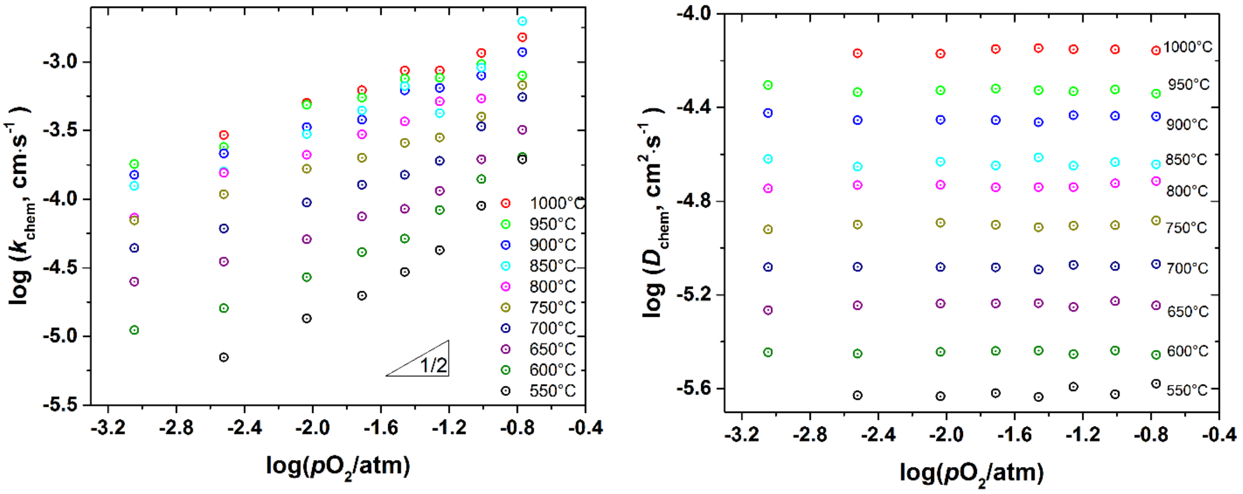

(**a**) (**b**)

Figure S12. The chemical surface exchange coefficient (**a**) and the chemical diffusion coefficient (**b**) of PBCF4 vs. *p*O2.

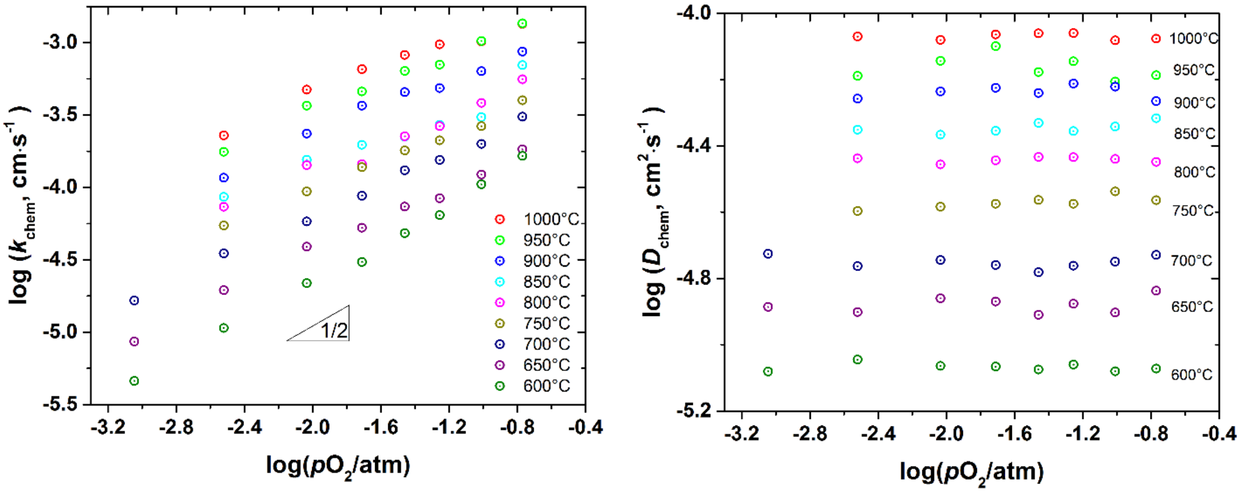

(**a**) (**b**)

Figure S13. The chemical surface exchange coefficient (**a**) and the chemical diffusion coefficient (**b**) of PBCF6 vs. *p*O_2.

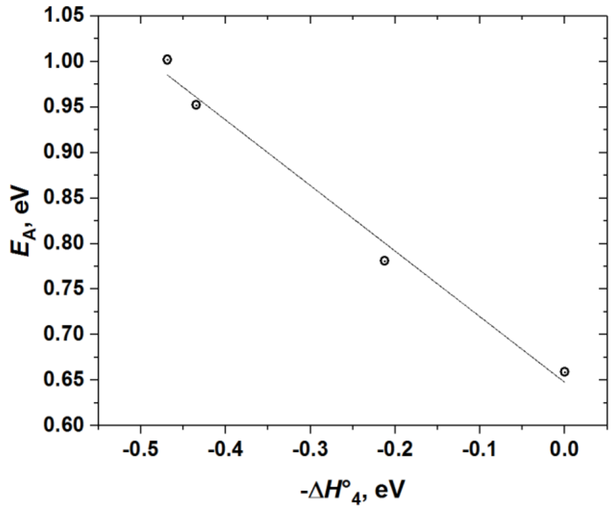

Figure S14. The activation energy of self-diffusion vs. the enthalpy of oxygen vacancies' localization in the Pr-layers for the PBCF oxides with different iron content (see also Table 2 and Figure 14 in the main text).
